# Impact of Glucagon-like Peptide-1 Receptor Agonists (GLP-1 RAs) on Increased Residual Gastric Content in Patients With and Without Concurrent Colonoscopy: A Retrospective Case–Control Study

**DOI:** 10.3390/jcm15062121

**Published:** 2026-03-10

**Authors:** Shiyi Chang, Yan Tang, Meng Wang, Shengjun Zhu, Xi Tan, Xiaowei Fan, Liping Lu, Bensong Duan, Li Shen

**Affiliations:** Endoscopy Center, Department of Gastroenterology, Shanghai East Hospital, School of Medicine, Tongji University, Shanghai 200120, China; 2532136@tongji.edu.cn (S.C.); tangyan0120@126.com (Y.T.); 17621607083@163.com (M.W.); 13816994954@163.com (S.Z.); 13262610990@163.com (X.T.); m15821873312@163.com (X.F.); 13564862656@163.com (L.L.)

**Keywords:** glucagon-like peptide-1 receptor agonists, residual gastric content, esophagogastroduodenoscopy, colonoscopy, bowel preparation, procedure abortion

## Abstract

**Background/Objectives**: The use of GLP-1 RAs has dramatically increased with expanded indications for diabetes mellitus and obesity. Delayed gastric emptying due to these medications can lead to increased residual gastric content (RGC). While previous studies have focused on Esophagogastroduodenoscopy (EGD), few have specifically analyzed the impact of GLP-1 RAs on residual gastric content in patients undergoing concurrent colonoscopy with adequate bowel preparation. **Methods**: A retrospective, case–control study was conducted at Shanghai East Hospital from January 2023 to June 2025. Adult patients with increased RGC were identified as cases. Controls without increased RGC were randomly selected at a 1:2 ratio, matched for age and sex. Multivariable logistic regression was used to assess the independent association between GLP-1 RAs use and increased RGC. **Results**: Among 131,255 procedures screened, 3746 patients were included (1257 with increased RGC and 2489 controls). GLP-1 RAs users had higher odds of increased RGC in both unadjusted [OR 15.20 (95% CI 5.98–38.61)] and adjusted analyses [aOR = 13.31 (95% CI 5.07–34.93)]. Other significant risk factors for RGC included diabetes-related complications [aOR = 8.89 (3.15–25.12)]. Interestingly, among the enrolled patients who used GLP-1 RAs and underwent concurrent colonoscopy, 19 of the 22 patients (86.4%) exhibited increased RGC, whereas only 3 (13.6%) did not. **Conclusions**: Perioperative use of GLP-1 RAs is associated with an increased residual gastric content in patients undergoing EGD alone or with concurrent colonoscopy. There was no aspiration event related to residual gastric content. Our study highlights the need for vigilant preoperative assessment and individualized periprocedural management in patients on GLP-1 RAs undergoing endoscopic procedures, despite having standardized adequate bowel preparation.

## 1. Introduction

Glucagon-like peptide 1 (GLP-1) is a peptide hormone secreted predominantly by the distal small intestine and colon that can reduce blood glucose by stimulating insulin release, slowing gastric emptying, inhibiting food intake, glucagon secretion, and modulating rodent β-cell proliferation [1]. GLP-1 receptor agonists (GLP-1 RAs) are analogs of the native human GLP-1 hormone that act on multiple pathophysiological defects in type 2 diabetes mellitus (T2DM), providing effective glycemic control, weight loss [2], a low risk of hypoglycemia, and cardiovascular [3] and renal benefits [4].

Besides the well-known hypoglycemic effect of GLP-1 RAs, increasing evidence showed that perioperative GLP-1 RAs use also profoundly affects the gastrointestinal motor system [5], slows gastric emptying, and reduces bowel motility due to inhibition of peristalsis at the level of GLP-1 receptors localized to myenteric neurons [6], which may increase the residual gastric content (RGC) and gastrointestinal symptoms. To date, several case reports [6,7,8,9] indicated that perioperative use of GLP-1 RAs was associated with increased RGC and intraoperative pulmonary aspiration [10,11,12] among patients undergoing Esophagogastroduodenoscopy (EGD). GLP-1 RAs are associated with adverse gastrointestinal effects such as nausea, vomiting, and delayed gastric emptying [13]. The presence of adverse gastrointestinal symptoms (nausea, vomiting, dyspepsia, abdominal distension) in patients taking GLP-1 RAs is predictive of increased RGC [14].

With the increased use of GLP-1 RAs and their delayed gastric emptying effect, the optimal management of these patients prior to EGD procedures has been discussed recently. Society recommendations vary between the Anesthesiology and Gastroenterology communities with respect to the timing of GLP-1 RA cessation prior to scheduled endoscopies. The American Society of Anesthesiologists (ASA) recommends stopping daily-dosed GLP-1 RAs on the day of the procedure and weekly-dosed GLP-1 receptor agonists a week prior to the procedure [15]. Several guidelines recommended withholding GLP-1 RAs before surgical procedures [16,17] due to the increased risk for regurgitation and pulmonary aspiration of gastric contents during deep and general anesthesia. In contrast, the 2024 American Gastroenterology Association (AGA) Rapid Clinical Practice Update advocates for a more individualized approach. It suggests that for patients on GLP-1 RAs who have adhered to standard fasting instructions (typically 8 h for solids and 2 h for liquids) and do not exhibit gastrointestinal symptoms (e.g., nausea, vomiting), proceeding with endoscopy is reasonable [18]. If gastrointestinal symptoms exist, assessing gastric emptying through solid tests or employing gastric ultrasound to detect retained contents may be warranted.

Current data regarding the discontinuation of GLP-1 RAs use in the periprocedural timeframe are conflicting and insufficient, which may result in the unnecessary abortion of endoscopic procedures, delaying medical care, and additional procedural costs or resource consumption. The association between GLP-1 RAs use and increased residual gastric content during gastrointestinal endoscopy remains unclear due to a lack of high-level evidence. Hence, we established a retrospective case–control study to investigate the association between GLP-1 RAs use and increased RGC in EGD with and without concurrent colonoscopy.

## 2. Materials and Methods

### 2.1. Study Design and Setting

**Figure 1:**
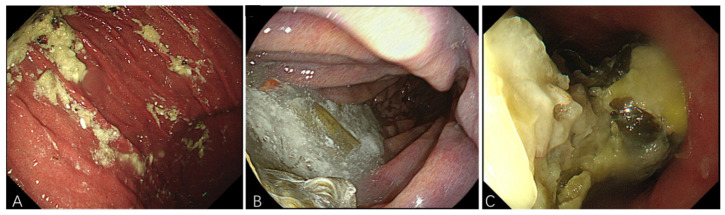
EGD images showing the amount of increased RGC as visually estimated by endoscopists: (**A**) small, (**B**) medium, (**C**) large.

This study was approved by the Ethics Committee at Shanghai East Hospital (Shanghai, China; approval No: 2025YS-152; June 2025). To identify eligible procedures, we searched the internal endoscopy database of Shanghai East Hospital for adult patients (older than 18 years) who had increased RGC presenting for EGD procedures alone or with concurrent colonoscopy at the endoscopy center in Shanghai East Hospital between January 2023 and June 2025. All endoscopic procedures were performed using standard video endoscopes, including the UHD-GT300T (Shanghai Aohua Endoscopy Co., Ltd., Shanghai, China), GIF-H290, GIF-HQ290, and GIF-H260 (Olympus Corporation, Tokyo, Japan), and EG-760R and EG-760Z (Fujifilm Corporation, Tokyo, Japan). Procedure reports were subsequently reviewed by 2 independent researchers to verify the presence of increased retained gastric content encountered during endoscopy. Increased RGC was defined as meeting either of the following criteria [19]: (1) Presence of any solid content from the esophagus to the pylorus. (2) Fluid content volume > 0.8 mL/kg as measured from the aspiration/suction canister (given its higher risk of aspiration). The amount of increased RGC was visually estimated (small/medium/large) by three experienced endoscopists by reviewing endoscopy reports and images (Figure 1). We defined aborted procedures either as procedure cancellations or the need for repeat procedures in Medcare electronic endoscopic reports (Medcare, Qingdao, China). The GLP-1 RAs investigated in this study were liraglutide and semaglutide (including both oral and subcutaneous formulations). Other medications in this class, such as dulaglutide, lixisenatide, exenatide, and tirzepatide, were not used by the study cohort.

### 2.2. Study Population and Case–Control Definition

We utilized a retrospective case–control design categorized by the presence or absence of RGC. The Case Group comprised patients with endoscopically identified increased RGC, while the Control Group consisted of patients with no residual content. To ensure statistical efficiency given the high volume of negative screenings, controls were randomly selected and frequency-matched to cases (1:2 ratio) by age and sex. A 1:2 ratio was chosen to maximize statistical power while maintaining computational efficiency in the conditional logistic regression model. For GLP-1 RA users, detailed pharmacological data—including indication, duration, and interruption interval—were retrieved from electronic records.

The preprocedural diet in our hospital system requires fasting from solid food for 8 h and clear liquids for 2 h prior to the procedure time. All patients were advised and adhered to preprocedural diets as dictated in the ASA guidelines. Patients who were undergoing concomitant colonoscopy received preoperative colonoscopy preparation. Patients who underwent concurrent colonoscopy were instructed to start a restricted, clear-liquid diet the day before their procedure and reduce their intake of high-fiber foods (e.g., fruits and vegetables) for 3 days prior to surgery. All endoscopy findings were reported by trained gastroenterologists who carried out the endoscopies. The presence of increased residual gastric content was taken as the study endpoint.

### 2.3. Exposure and Covariates

The following demographic information was collected from electronic health records: age at time of procedure, sex, body mass index (BMI), and the presence of medical conditions potentially associated with increased RGC (diabetes mellitus, diabetes complications, obesity, metabolic syndrome, reflux esophagitis). The following endoscopic data were extracted: anesthesia type, inpatient vs. outpatient status, afternoon procedure time, and the presence or absence of increased RGC. All endoscopy images were digitally recorded and stored in the institutional database. The RGC volume was routinely visually (small, medium, or large) [14,20,21] estimated by the endoscopist.

### 2.4. Exclusion Criteria

The exclusion criteria of our study included emergency procedures that took place without fasting, patients who were pregnant, patients with a history of gastroparesis and acute conditions known to cause gastroparesis (such as acute gastrointestinal bleeding and acute pancreatitis), patients who had received gastrointestinal or other abdominal surgery (such as prior gastric resection, banding or bypass), patients with structural foregut abnormalities (esophageal, gastric or duodenal stricture/stenosis, including esophageal strictures, hiatal hernias, pyloric obstruction, intestinal spasms, achalasia, and Crohn’s disease), patients who had medications associated with delayed gastric emptying (opioids, antacids, antiemetics, anticholinergics, cardiovascular medications) and incomplete data recording due to varying reasons.

### 2.5. Statistical Analysis

The normal quantile-quantile plot was used to assess data distribution normality. Characteristics of patients with and without increased RGC on EGD were compared using the Mann–Whitney U test for nonnormally distributed continuous variables and χ^2^ or Fisher’s exact test for categorical variables, as appropriate. Categorical variables are presented as frequencies and percentages, while continuous variables are presented as means and SDs for normally distributed variables and median and interquartile range for non-normally distributed variables. Univariate logistic regression analyses were used to determine associations between baseline demographic factors and primary outcomes. Univariable associations were first assessed, and crude odds ratios (ORs) and 95% confidence intervals (CIs) are presented in the corresponding Supplemental Materials. To account for the simultaneous impact of several measured predictors, a conditional logistic regression model was fitted. Prior to multivariable modeling, the absence of multicollinearity among the covariates was confirmed using the Variance Inflation Factor (VIF), with all variables demonstrating a VIF of less than 2. The results of multivariable analysis are expressed as adjusted ORs with 95% CIs. Variables demonstrating statistical significance in the univariate analysis, along with pre-specified clinically relevant confounders (e.g., DM, afternoon procedure), were included in the multivariable conditional logistic regression model. All significance tests were 2-sided, and a *p*-value less than 0.05 was considered statistically significant. All analyses were performed using R software version 4.4.3 (R Foundation for Statistical Computing, Vienna, Austria).

## 3. Results

**Figure 2:**
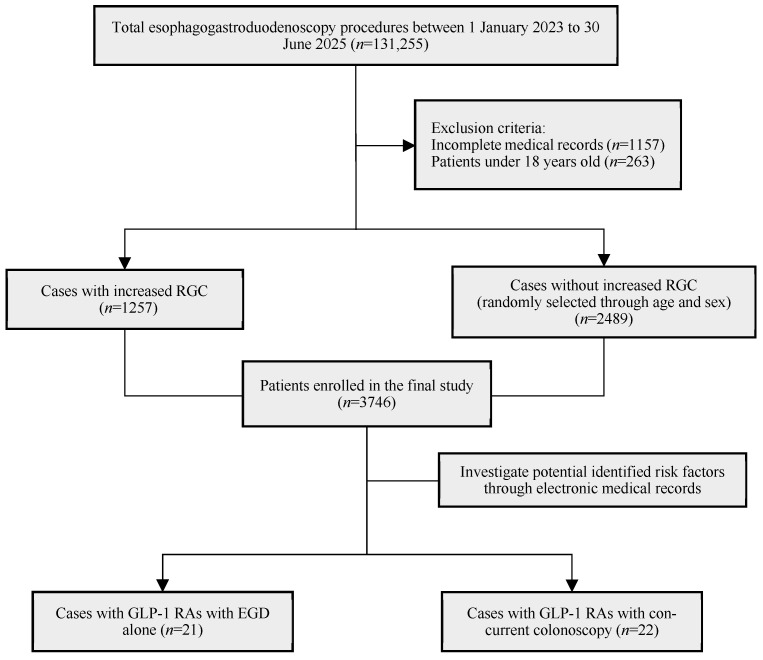
Flowchart of patient selection. RGC, residual gastric content; EGD, esophagogastroduodenoscopy.

Of 131,255 patients initially identified, 3746 patients were included in the final analysis after removal of duplicate procedures and exclusion of patients with incomplete comorbidity or medication data (Figure 2). This cohort comprised 1257 patients with increased RGC and 2489 age- and sex-matched patients without increased RGC.

Table 1 shows the demographic and clinical characteristics of the study cohort. The median age of patients was 61.00 [45.00, 70.00] years, 2236 (59.7) were male, 3021 (80.6%) received anesthesia, and 1487 (39.7%) received inpatient endoscopy (Table 1). The unadjusted analysis showed that DM complications [OR = 9.05 (95% CI 3.44–23.79)], metabolic syndrome [OR = 7.00 (95% CI 1.45–33.69)] were associated with increased RGC. The usage of GLP-1 RAs (43 patients in total, 38 on semaglutide and 5 on liraglutide) was associated with a relatively high risk of increased RGC during EGDs [OR 15.20 (95% CI 5.98–38.61)]. In univariate analysis, obesity (defined as BMI ≥ 28 kg/m^2^) was an important predictor of increased RGC [OR = 2.04 (95% CI 1.49–2.79)]. However, when BMI was analyzed as a continuous variable in a multivariate model, it demonstrated a weak inverse association with increased RGC [OR = 0.88 (95% CI 0.86–0.91)]. In our study, procedures were aborted in 3.87% (145/3746) of all enrolled patients. Notably, this outcome was markedly more frequent among the 1257 patients with increased RGC, with an incidence of 10.02% (*n* = 126).

To account for potential confounding effects, a multivariable conditional logistic regression model was fitted. The final model included all variables that demonstrated statistical significance (*p* < 0.05) in the univariate analysis (i.e., anesthesia, hypertension, DM complications, obesity, metabolic syndrome, and reflux esophagitis), alongside pre-specified clinically relevant confounders (diabetes mellitus and afternoon procedure). As noted in Table 2, GLP-1 RAs use was noted to be associated with high rates of increased RGC [aOR = 13.31 (95% CI 5.07–34.93)]. Patients with DM complications [aOR = 8.89 (3.15–25.12)] had a significantly higher likelihood of increased RGC compared to those without these conditions. None of the patients with increased RGC experienced an aspiration event.

Interestingly, among the enrolled patients who received GLP-1 RAs and underwent concurrent colonoscopy, 19 out of 22 (86.4%) exhibited increased RGC on EGDs, whereas only 3 (13.6%) did not (Table 3; individual characteristics are detailed in Supplementary Table S1). Notably, all of these 19 patients with increased RGC were also found to have solid stool during the concurrent colonoscopy. This result indicates that, despite standard preoperative bowel preparation for colonoscopy, a substantial proportion of patients undergoing concomitant procedures still developed varying degrees of increased RGC, which subsequently led to aborted procedures and repeated examinations.

## 4. Discussion

Our retrospective case–control study demonstrates the potential association between the usage of GLP-1 receptor agonists (GLP-1 RAs) and an increased risk of residual gastric content (RGC) during esophagogastroduodenoscopy (EGD), particularly in patients with diabetic complications and concurrent colonoscopy.

Although one study reported that GLP-1 RAs did not significantly increase odds of retained food on EGD [22], our findings align with most existing studies demonstrating that GLP-1 RAs therapy elevates the risk of gastric residue, which is further amplified by the presence of diabetic complications. After adjusting for potential confounders, patients using GLP-1 RAs had more than a 13-fold increase in having increased RGC compared to non-users. This finding is consistent with the known physiological effects of GLP-1 RAs, which include delayed gastric emptying and reduced gastrointestinal motility mediated by GLP-1 receptors on myenteric neurons [6,8]. This risk is exacerbated in patients with T2DM complications [23], who demonstrated an odds ratio (OR) of 8.89 (95% CI: 3.15–25.12), suggesting a possible contribution from diabetes-mediated neuropathy impacting gastric motility.

Previous studies have demonstrated that both higher HbA1C levels and pre-operative glucose levels can, in and of themselves, be associated with retained gastric contents [24,25]. A cross-sectional study found that for every 1% increase in HbA1C, there was 36% increase in the odds of retained food. Uncontrolled T2DM and/or DM complications, in addition, were statistically significant risk factors of gastric retention, supporting our approach. A risk-stratification approach is recommended, with particular attention to patients with uncontrolled diabetes or DM complications. For these high-risk individuals, a multidisciplinary evaluation is essential to balance procedural risks with clinical needs. Preprocedural measures, including the adoption of a clear liquid diet, should be strongly considered to mitigate potential complications.

In contrast to previous reports suggesting a protective effect of bowel preparation against gastric residue in GLP-1 RA users [14,23,26], our findings demonstrate that increased gastric residue persists even after such preparation in patients undergoing EGD combined colonoscopy. In our GLP-1 RA subgroup, increased gastric residue was observed in 86.4% of patients undergoing combined EGD–colonoscopy, and all of these patients also had solid stool on colonoscopy, despite standard bowel preparation. Although the small sample size and lack of a non–GLP-1 RA comparison group within this subgroup warrant cautious interpretation, these findings suggest that conventional bowel preparation alone may not be sufficient to offset the gastric-retentive effects of GLP-1 RAs. We hypothesize that this discrepancy arises because the purgative agents (e.g., 4 L polyethylene glycol) used for bowel preparation act primarily in the colon and do not enhance gastric emptying [27]. Furthermore, we hypothesize that this discrepancy arises from the specific pharmacodynamics of GLP-1 RAs, which induce a “functional obstruction” by inhibiting antral contractility and increasing pyloric tone [28,29]. GLP-1 RAs suppress the interdigestive migrating motor complex (MMC) [29], particularly the phase III activity responsible for clearing indigestible solids and fluids during fasting. Based on our endoscopic observations, the residual gastric content in the GLP-1 RA cohort was predominantly solid. Because GLP-1 RAs severely impair the antral grinding and MMC sweeping required for solid emptying, we hypothesize that the large volume of liquid PEG essentially bypassed the solid contents and emptied into the duodenum, failing to provide the mechanical ‘washout’ effect typically seen in patients with altered gastric anatomy (e.g., partial gastrectomy). This observation aligns with our multivariable analysis, which identifies GLP-1 RAs as a dominant independent risk factor for increased RGC (aOR 13.31; 95% CI 5.07–34.93) even after adjusting for periprocedural factors.

The presence of gastric residue can lead to poor mucosal visualization, compromising diagnostic accuracy, and may interfere with therapeutic interventions. Prior studies reported that the rates of procedure termination or incomplete examination ranged from 2% to 30% due to gastric residue, with higher volumes of residue correlating strongly with procedural incompletion [30,31]. One study reported that 9.2% of patients with gastric residue on upper endoscopy required a repeat procedure within 30 days due to incomplete examination [30]. In our study, procedures were aborted in 3.87% (145/3746) of all enrolled patients, with a significantly higher rate of 10.02% (126/1257) observed among those with increased RGC.

A previous retrospective study from Mayo Clinic Rochester identified only two definite episodes of pulmonary aspiration among 4134 endoscopic procedures, representing 4.8 cases per 10,000 endoscopies [32]. The results are similar to another study in which the incidence of gastric-to-pulmonary aspiration in 60,770 patients undergoing elective upper GI endoscopy from 2010 to 2016 was 4.6 cases per 10,000 endoscopies [33]. The presence of gastric residue confers a minimal risk for adverse respiratory events, with reported rates ranging from 0.2% to 3.5% [30,34]. There was only one case of pulmonary aspiration in the group receiving semaglutide [14]. In our study, we did not encounter any adverse respiratory events or aspiration in our study patients, further verifying the relatively low risk of pulmonary complications with GLP-1 RAs use. As for the current ubiquity of GLP-1 RAs use for patients with DM and/or obesity, it is also expected that aspiration events will occur. Given the rarity and severity of pulmonary aspiration, we still recommend adopting a conservative approach to safety in the absence of clear data, which includes the utilization of preoperative gastric ultrasound in guiding individualized airway management or the delay of elective procedures.

The results of our study challenge the recommendations of the American Society of Anesthesiologists (ASA) and validate concerns of gastroenterology societies. ASA advocated for a liberal approach: if the patient has no gastrointestinal symptoms and the GLP-1 RAs have been held as advised, it is recommended to proceed as usual. Our findings demonstrated a relatively high increase in RGC, even in patients who received concurrent colonoscopies, which led to a 10.02% rate of procedural cancellation. Although no aspiration events were observed, the high rate of aborted procedures underscores that a symptom-triggered approach is insufficient. We advocate for a more stringent preoperative management strategy for preoperative GLP-1 RA interruption in patients with or without colonoscopies to prevent procedure cancellations and mitigate the underlying risk. Although there is no consensus about preparation for gastric residue in an EGD among the high-risk group now, it was reported that metoclopramide [35], domperidone [36], and erythromycin [37] accelerated gastric emptying. Therefore, if retained gastric contents are identified on ultrasound prior to the procedure, a more individualized preoperative plan is needed. It is reasonable to use IV erythromycin (∼3 mg/kg) [37] or have a longer fasting time and then reassess gastric content with ultrasound prior to commencing the procedure.

Several limitations of the present study should be acknowledged. First, this was a single-center retrospective study, and the number of patients exposed to GLP-1 RAs was relatively small, which limits the precision of the estimated effect and the ability to perform more granular subgroup analyses. Therefore, a further multicenter study is required. Second, the wide confidence interval for the odds ratio of GLP-1 RAs reflects this limited sample size and the low event rate in the control group, indicating imprecision in the point estimate. Third, data on GLP-1 RAs use were collected via electronic medical records, which were limited in capturing prescriptions from external healthcare systems. Fourth, there remains the possibility that prescriptions from other hospitals affect gastrointestinal motility, such as concomitant medication use (e.g., opioids), which could have influenced the results. Fifth, the definition of RGC was based on endoscopic visualization rather than objective measures such as gastric volume ultrasound, which may introduce subjectivity.

## 5. Conclusions

In conclusion, we found the association between GLP-1 RA use and increased residual gastric content during EGD. The relatively high prevalence of increased RGC underscores the need for heightened preoperative assessment and possibly more prolonged fasting or drug withholding in GLP-1 RAs users. Increased RGC remained evident in patients despite the completion of the preoperative standard bowel preparation protocol. Notably, most of the GLP-1 RAs users had solid stools on concurrent colonoscopy. Our results also reinforce the role of other patient-related factors in RGC risk. Diabetes complications and metabolic syndrome were all potentially associated with increased RGC, highlighting the multifactorial nature of gastric retention in patients undergoing upper endoscopy with or without concurrent colonoscopy.

## Figures and Tables

**Table 1:** Comparison of demographics and clinical characteristics of patients according to the presence/absence of increased RGC.

	Increased Residual Gastric Content			Unadjusted OR (95% CI)	*p*-Value
	Total (*n* = 3746)	RGC (*n* = 1257)	non-RGC (*n* = 2489)		
**Age ^a^** (year)	61.00 [45.00, 70.00]	61.00 [45.00, 70.00]	61.00 [45.00, 70.00]		
**Sex ^b^**					
**Male**	2236 (59.7)	749 (59.6)	1487 (59.7)		
**Female**	1510 (40.3)	508 (40.4)	1002 (40.3)		
**BMI ^a^ (kg/m^2^)**	22.65 [20.96, 24.57]	21.83 [20.20, 23.81]	23.11 [21.45, 24.80]	0.88 (0.86–0.91)	<0.001 ***
**Anesthesia ^b^**	3021 (80.6)	859(68.3)	2162(86.9)	0.32 (0.27–0.38)	<0.001 ***
**Hospitalization ^b^**					
inpatient	1487 (39.7)	506 (40.3)	981 (39.4)	1.04 (0.89–1.20)	0.6043
outpatient	2259 (60.3)	751 (59.7)	1508 (60.6)		
**Afternoon procedure ^b^**	677 (18.1)	224 (17.8)	453 (18.2)	0.97 (0.81–1.16)	0.7861
**Hypertension ^b^**	940 (25.1)	224 (17.8)	716 (28.8)	0.51 (0.43–0.61)	<0.001 ***
**DM ^b^**	522 (13.9)	178 (14.2)	344 (13.8)	1.02 (0.84–1.24)	0.8139
**DM complications ^b^**	29 (0.8)	24 (1.9)	5 (0.2)	9.05 (3.44–23.79)	<0.001 ***
**Obesity ^b^**	172 (4.6)	85 (6.8)	87 (3.5)	2.04 (1.49–2.79)	<0.001 ***
**Metabolic syndrome ^b^**	9 (0.2)	7 (0.6)	2 (0.1)	7.00 (1.45–33.69)	0.0152 *
**Reflux esophagitis ^b^**	787 (21.0)	227 (18.1)	560 (22.5)	0.75 (0.63–0.89)	0.0016 **
**GLP-1 RAs ^b^**	43 (1.1)	38 (3.0)	5 (0.2)	15.20 (5.98–38.61)	<0.001 ***
Semaglutide	38 (1.0)	34 (2.7)	4 (0.2)	17.00 (6.03–47.90)	<0.001 ***
Liraglutide	5 (0.1)	4 (0.3)	1 (0.0)	8.00 (0.89–71.57)	0.0629

**BMI**, body mass index; **DM**, diabetes mellitus; **DM complications**, including diabetic nephropathy, diabetic retinopathy, diabetic peripheral neuropathy; **OR**, odds ratio; **CI**, confidence interval. *p*-values are based on regression analysis. * *p* < 0.05, ** *p* < 0.01, *** *p* < 0.001. ^a^ Values expressed as median [percentile 25–75%]. ^b^ Values expressed as *n* (%).

**Table 2:** Multivariable conditional logistic regression analyses of increased RGC.

	Adjusted OR (95% CI)	*p*-Value
**GLP-1 RAs**	13.31 (5.07–34.93)	<0.001 ***
**DM complications**	8.89 (3.15–25.12)	<0.001 ***
**Obesity**	2.02 (1.43–2.85)	0.003 **
**Anesthesia**	0.32 (0.27–0.39)	<0.001 ***
**Hypertension**	0.48 (0.40–0.59)	<0.001 ***
**Metabolic syndrome**	7.11 (1.30–38.83)	0.024 *
**Reflux esophagitis**	0.78 (0.65–0.94)	0.011 *
**Afternoon procedure**	0.88 (0.72–1.07)	0.198
**DM**	1.07 (0.84–1.37)	0.564

**DM complications**, including diabetic nephropathy, diabetic retinopathy, diabetic peripheral neuropathy; **Obesity**, defined as BMI ≥ 28 kg/m^2^; **OR**, odds ratio; **CI**, confidence interval. The multivariable model was adjusted for all covariates demonstrating statistical significance (*p* < 0.05) in the univariate analysis (i.e., obesity, anesthesia, hypertension, metabolic syndrome, and reflux esophagitis), as well as pre-specified clinically relevant confounders (DM and Afternoon procedure). Absence of significant multicollinearity among these variables was confirmed using the Variance Inflation Factor (VIF). No significant multicollinearity was detected (all VIF < 2). *p < 0.05, ** *p* < 0.01, *** *p* < 0.001.

**Table 3:** Clinical characteristics and procedural findings of GLP-1 RAs users undergoing concurrent colonoscopy, *n* = 22.

Category	Characteristic	Value
**Demographics**	Age (years)	61.0 [45.0, 70.0]
	Sex (Male)	13 (59.1%)
**Primary outcomes**	Increased RGC	19 (86.4%)
**Procedural impact**	Aborted/repeated procedures	19 (86.4%)
	Solid stool on concurrent colonoscopy	19 (86.4%)
**GLP-1 RA profile**	**Type of Agent**	
	Semaglutide	19 (86.4%)
	Liraglutide	3 (13.6%)
	**Indication**	
	Diabetes management	14 (63.6%)
	Weight loss	8 (36.4%)
	Median duration of use (months)	6.0 [2.25, 6.0]
	Median interruption interval (days)	3.5 [3.0, 4.75]

**RGC**, residual gastric content; **GLP-1 RA**, glucagon-like peptide-1 receptor agonist; Increased RGC was defined as the presence of any solid content or fluid volume > 0.8 mL/kg. **Interruption interval**, the time elapsed between the last dose of GLP-1 RA and the scheduled endoscopic procedure. Values are expressed as *n* (%) **or** median [interquartile range, 25–75%].

## Data Availability

The raw data supporting the conclusions of this article will be made available by the authors on request.

## Supplementary Materials

The following supporting information can be downloaded at: https://www.mdpi.com/article/10.3390/jcm15062121/s1. Table S1: Individual characteristics of GLP-1 RA users undergoing combined EGD and colonoscopy.

## Abbreviations

The following abbreviations are used in this manuscript:

GLP-1 RAs	Glucagon-like peptide-1 receptor agonists
RGC	Residual gastric content
EGD	Esophagogastroduodenoscopy
