# Supplementary material for: Impact of Glucagon-like Peptide-1 Receptor Agonists (GLP-1 RAs) on Increased Residual Gastric Content in Patients With and Without Concurrent Colonoscopy: A Retrospective Case–Control Study
Source: J Clin Med. 2026 Mar 10;15(6):2121. doi: 10.3390/jcm15062121 (PMC13027199; doi:10.3390/jcm15062121)
Supplement: Supplementary file 1 [file jcm-15-02121-s001.zip › jcm-4142097-supplementary.pdf]

Supplementary Table S1. Individual characteristics of GLP-1 RA users undergoing combined EGD and colonoscopy.

NO	Age	Sex	Obesity	DM	DM complications	GLP-1 RAs type	Increased RGC	Amount of increased RGC	Purpose of prescription	Time intervals of interruption (day)	Duration of GLP-1 RAs Use (month)
1	61	F	No	Yes	No	LG	Yes	large	GC	4	6
2	30	F	No	No	No	SG	Yes	large	WL	3	3
3	55	F	No	No	No	SG	Yes	small	WL	6	1
4	28	F	No	No	No	SG	Yes	large	WL	3	1
5	42	M	No	Yes	Yes	SG	Yes	large	GC	2	1
6	39	F	No	Yes	No	SG	Yes	large	GC	2	2
7	54	F	No	Yes	Yes	SG	Yes	large	GC	1	6
8	64	M	Yes	Yes	No	SG	Yes	small	GC	6	＞12
9	51	M	No	Yes	No	SG	Yes	large	GC	3	12
10	64	M	No	Yes	No	LG	Yes	medium	GC	4	1
11	66	F	No	Yes	No	SG	Yes	medium	GC	3	6
12	26	F	No	No	No	SG	Yes	small	WL	5	1
13	28	F	No	No	No	SG	Yes	large	WL	2	3
14	72	M	No	Yes	No	SG	Yes	small	GC	3	3
15	64	F	No	No	No	SG	Yes	large	WL	4	＞12
16	69	M	No	Yes	No	LG	Yes	medium	GC	5	6
17	32	F	No	No	No	SG	Yes	small	WL	5	6
18	32	M	No	No	No	SG	Yes	medium	WL	4	6
19	35	M	No	Yes	No	LG	Yes	large	GC	3	3
20	72	F	No	Yes	No	LG	No	-	GC	5	＞12
21	73	M	No	Yes	No	SG	No	-	GC	3	＞12
22	64	F	No	Yes	No	SG	No	-	GC	4	6

M, Male; F, Female; SG, Semaglutide; LG, Liraglutide; GC, Glycemic control; WL, Weight loss.
